# Supplementary material for: New Deep Learning Model to Estimate Ozone Concentrations Found Worrying Exposure Level over Eastern China
Source: Int J Environ Res Public Health. 2022 Jun 11;19(12):7186. doi: 10.3390/ijerph19127186 (PMC9223487; doi:10.3390/ijerph19127186)
Supplement: Supplementary file 1 [file ijerph-19-07186-s001.zip › ijerph-1752282-supplementary.pdf]

Figure

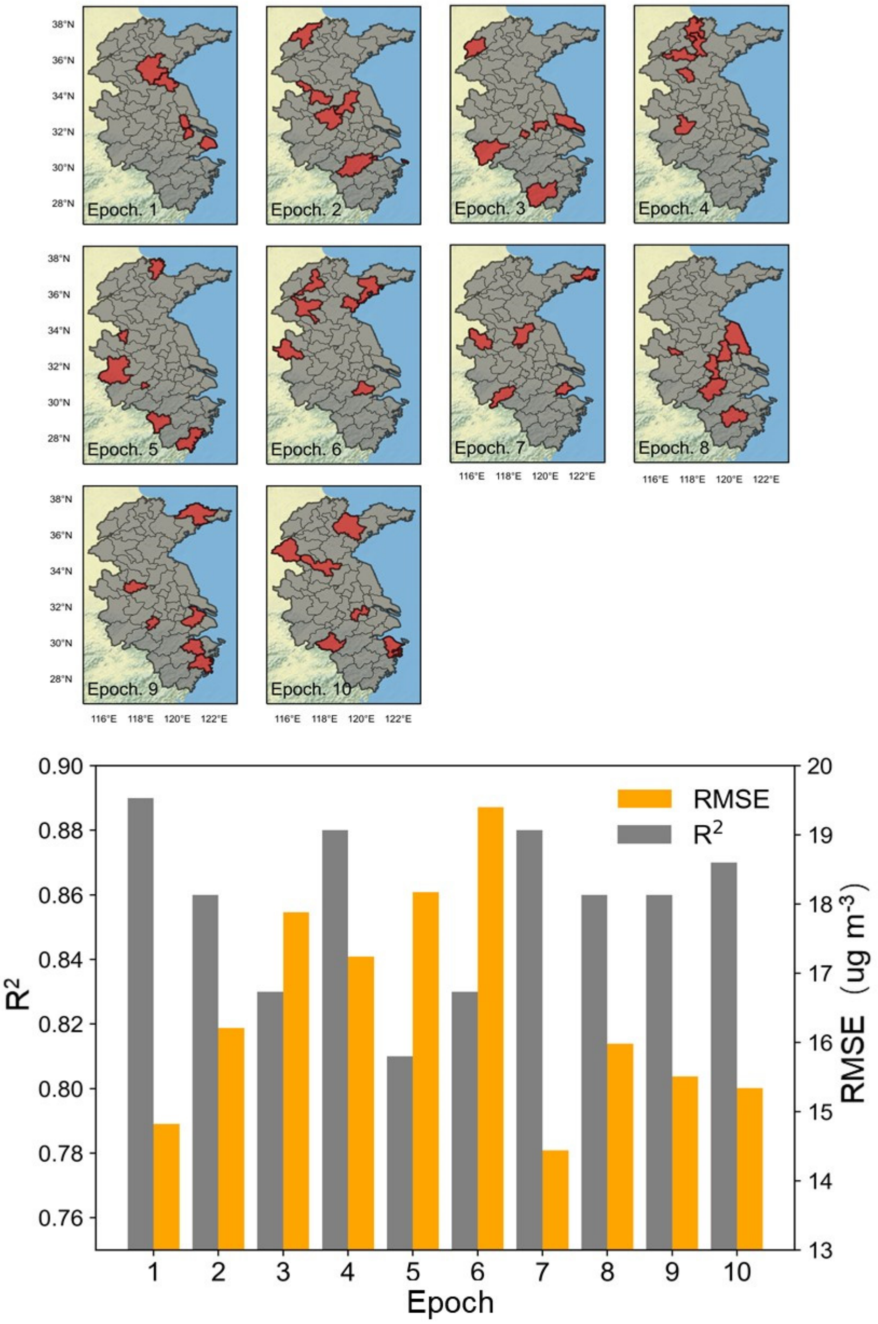

Figure S1 Sample division in the city-based CV10, reds are the testing cities and grays are the training cities.

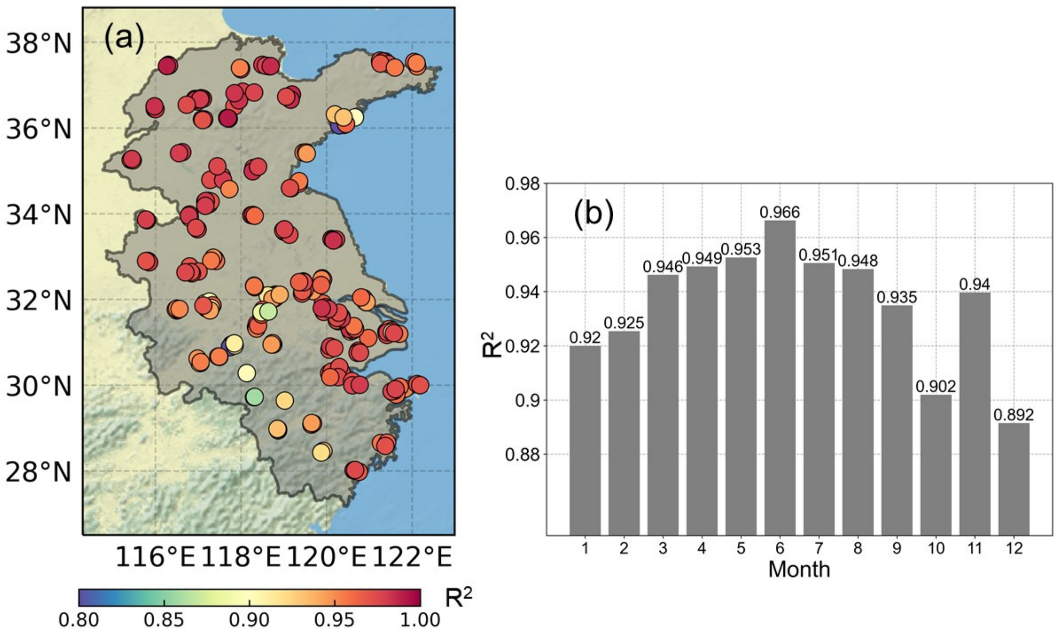

Figure S2. R^2 of generated data for each ground monitoring site (a) and each month (b).

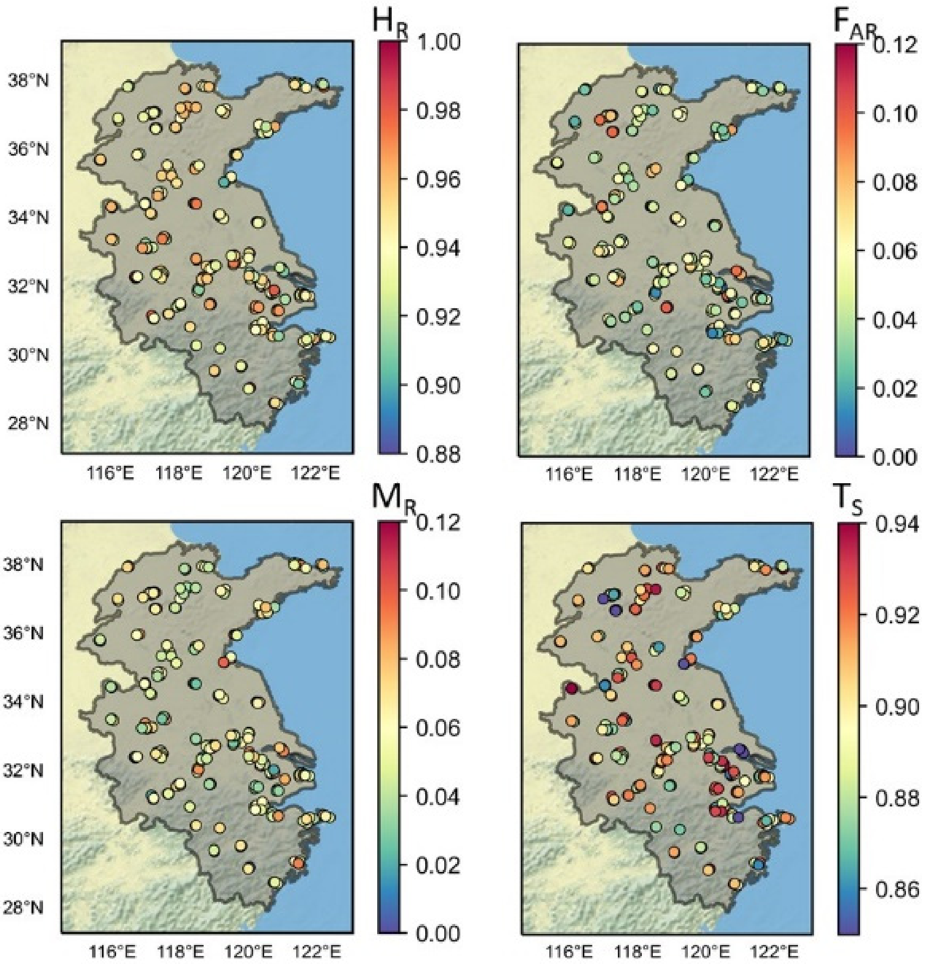

Figure S3. Peak validation results for each ground monitoring site

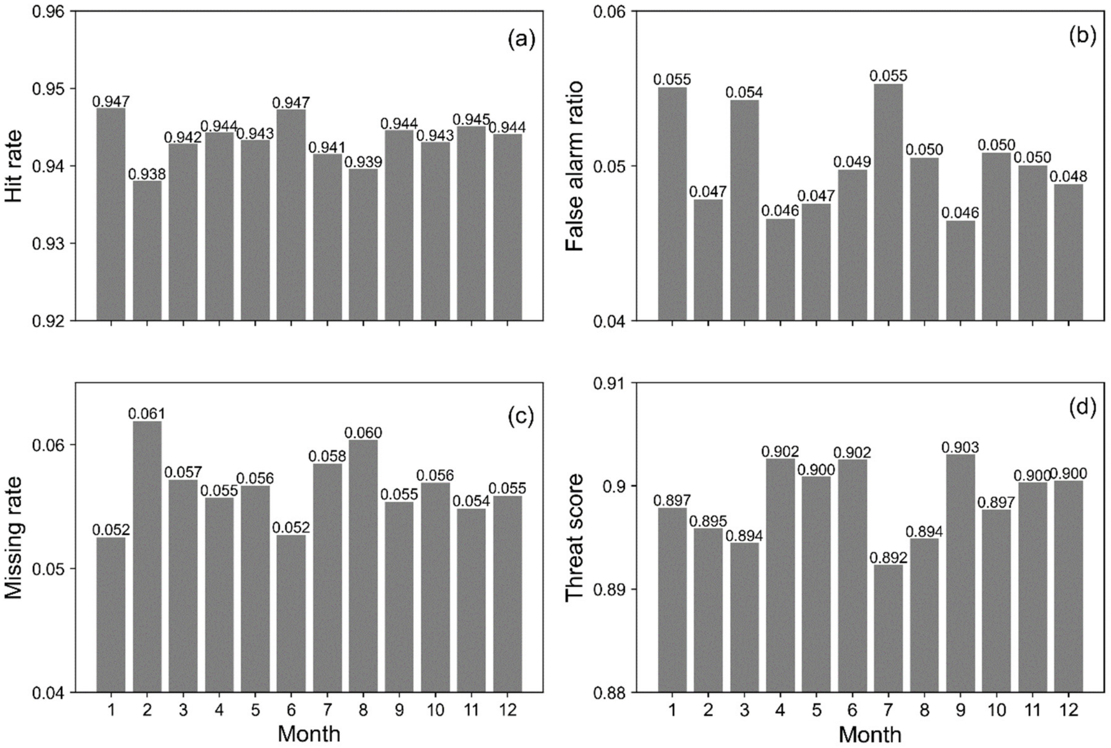

Figure S4. Monthly variations of peak validation metrics.

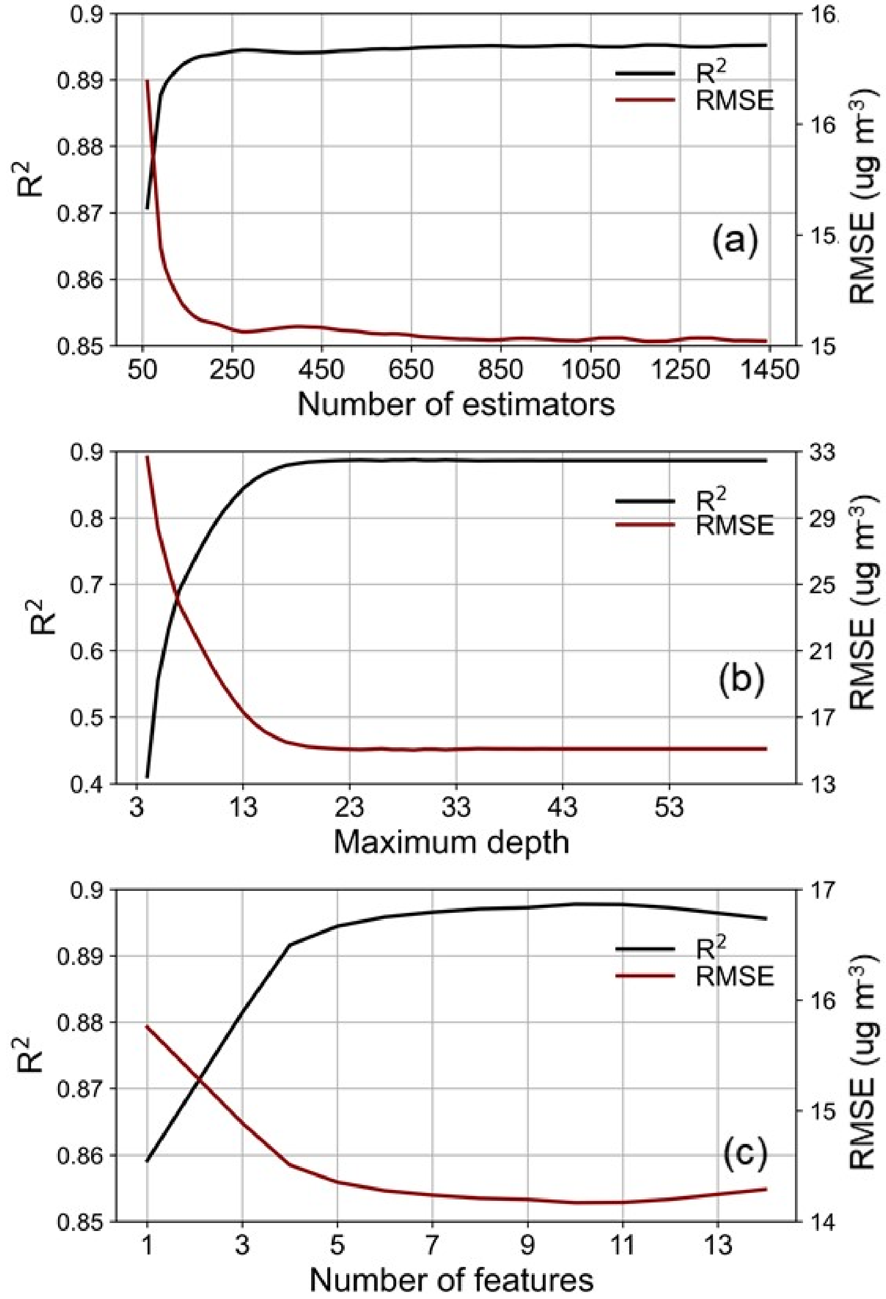

Figure S5. R^2 and RMSE curves of RF model with different parameters. The number of weak estimators directly determines the RF model fitting ability, which should be given priority to. It can be seen from Figure (a), RF model could be stabilized when the number of week estimators greater than ~700. The maximum depth and the number of features to consider affect the fitting ability of each weak estimator, and we finally determined 25 (Figure b) and 10 (Figure c), respectively

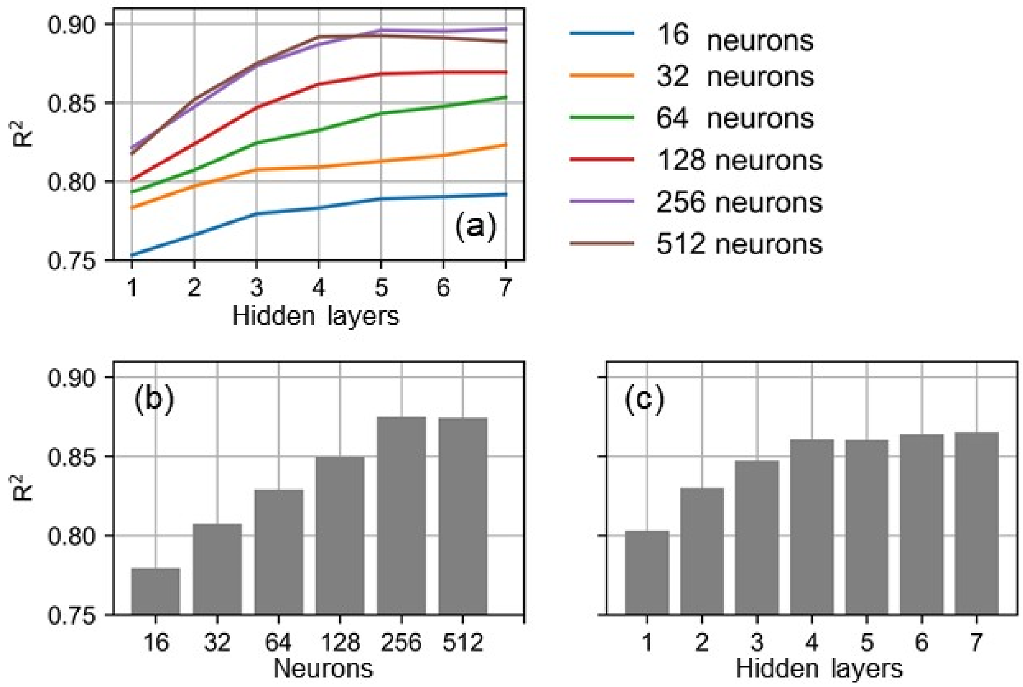

Figure S6. DNN model performance with different parameters. (a) is the R^2 curves of 16, 32, 64, 128, 256 512 neurons with different layers (b) is mean R^2 values of different neurons, and (c) is mean R^2 values of different hidden layers.

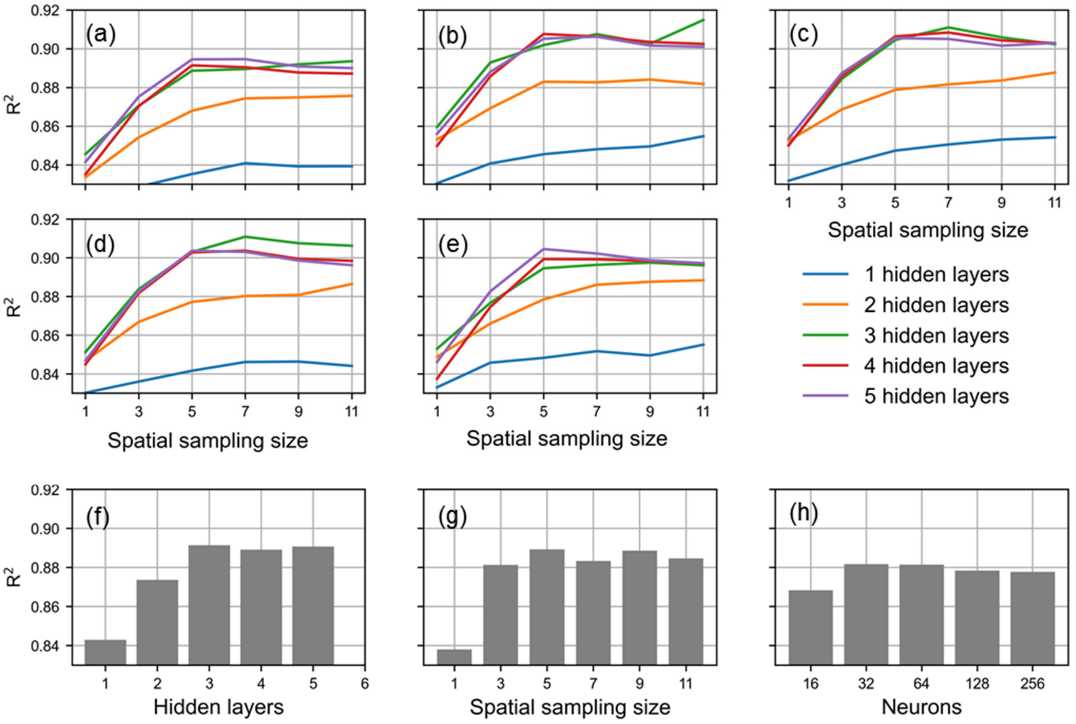

Figure S7. The spatial sampling size of CNN determines the amount of spatial information, so spatial sampling size replaces the time steps of AR-LSTM model as a hyperparameter. (a)-(e) are the R^2 curves of 16, 32, 64, 128, 256 neurons with different layers and spatial sampling size, respectively. (f)-(h) are mean values of different hidden layers, spatial sampling size and the numbers of neurons, respectively.

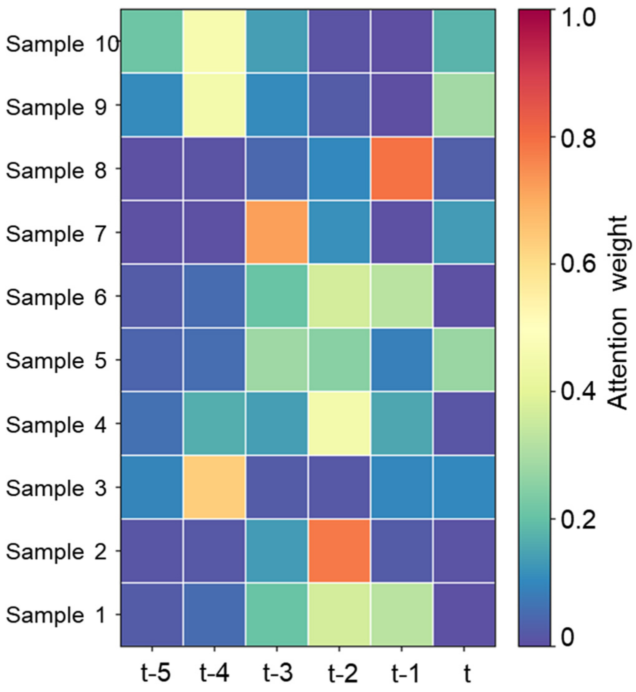

Figure S8. Some samples whose largest attention weights do not appear at the last epoch.

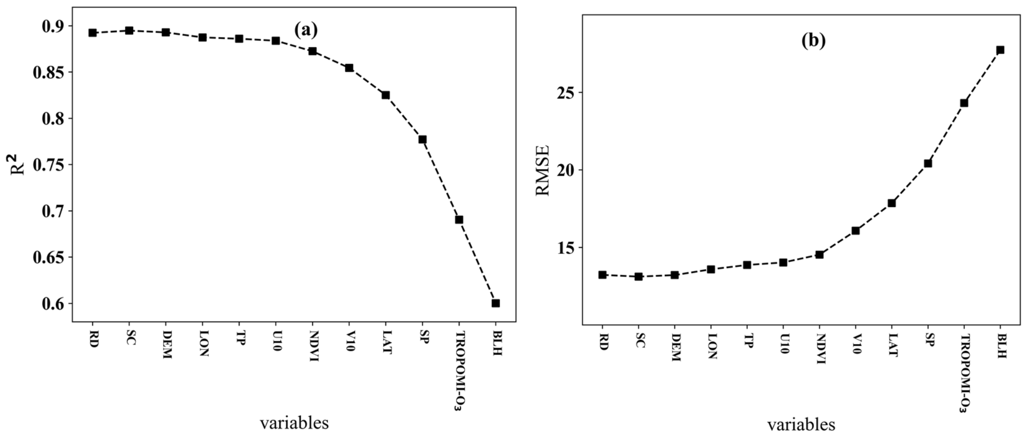

Figure S9. backward variable selection, the changes in R2 (a) and RMSE (b).

Table

Table S1. List of predictive variables and descriptions.

Symbol	Variable definition	Unit	Spatial resolution	Temporal resolution	Source
TROPOMI-O_3	TROPOMI-retrieve total O_3 column density	mol m^{-2}	5.5km × 3.5km	daily	Copernicus Sentinel Data Hub
SC	Surface classification	-		-	
DEM	Surface elevation latitude	m		-	
SSRD	Solar radiation downwards	J m^{-2}	0.25° × 0.25°	hourly	European Center for Medium-Range Weather Forecasts
D2M	2-m dew point temperature	K			
T2M	2-m temperature	K			
U10	10-m eastward wind component	m s^{-1}			
V10	10-m northward wind component	m s^{-1}			
BLH	Boundary layer height	m			
MSL	Mean sea level pressure	Pa			
SP	Surface pressure	Pa			
TP	Total precipitation	m			
RD	Road density	m km^{-2}	0.1° × 0.1°	-	Open Street
NDVI	Normalized difference vegetation index	-	500m × 500m	16 day	MODIS
LON	Longitude	°	-	-	-
LAT	Latitude	°			
DOY	Day of year	-			

Table S2. Comparison wtih other works.

Authors	Study region	Study period	Model	Spatial resolution	Performance[1]	
					R^2	RMSE
Li et al. [1]	Guangdong-Hong Kong-Macao Greater Bay Area	2018.12-2020.08	STE-ResNet	0.05°	0.93	12.99
Chen et al. [2]	China	2008-2019	Random forest	0.0625°	0.84	-
Wang et al. [3]	California	2018.05-2019.05	Random forest	10 km	0.84	-
Li et al. [4]	Hainan	2013.05-2017.12	Xgboost	0.1°	0.59	24.14
Wang et al. [5]	China	2020	Light-GBM	0.05°	0.91	14.14

[1] Samble-based CV.

References

1. Li, T., and Cheng, X. Estimating daily full-coverage surface ozone concentration using satellite observations and a spatiotemporally embedded deep learning approach. *Int. J. Appl. Earth Obs.* **2021**, 101, 102356. https://doi.org/10.1016/j.jag.2021.102356
2. Chen, G., Chen, J., Dong, G., Yang, B., Liu, Y., Lu, T., Yu, P., Guo, Y., and Li, S. Improving satellite-based estimation of surface ozone across China during 2008 – 2019 using iterative random forest model and high-resolution grid meteorological data. *Sustain. Cities Soc.* **2021**, 69, 102807. https://doi.org/10.1016/j.scs.2021.102807
3. Wang, W., Liu, X., Bi, J., and Liu, Y. A machine learning model to estimate ground-level ozone concentrations in California using TROPOMI data and high-resolution meteorology. *Environ. Int.* **2022**, 158, 106917. https://doi.org/10.1016/j.envint.2021.106917
4. Li, R., Cui, L., Fu, H., Li, J., Zhao, Y., and Chen, J. Satellite-based estimation of full-coverage ozone (O3) concentration and health effect assessment across Hainan Island. *J. Clean. Prod.* **2020**, 244, 118773. https://doi.org/10.1016/j.jclepro.2019.118773
5. Wang, Y., Yuan, Q., Li, T., Zhu, L., and Zhang, L. Estimating daily full-coverage near surface O3, CO, and NO2 concentrations at a high spatial resolution over China based on S5P-TROPOMI and GEOS-FP. *ISPRS-J. Photogramm. Remote Sens.* **2021**, 175, 311-325. https://doi.org/10.1016/j.isprsjprs.2021.03.018
